# Impact of Thermomechanical Aging on Marginal Fit and Fracture Resistance of CAD/CAM Endocrowns Fabricated from Different Materials

**DOI:** 10.3390/polym18010143

**Published:** 2026-01-05

**Authors:** Bülent Kadir Tartuk, Gizem Akın Tartuk

**Affiliations:** 1 Department of Prosthodontics, Faculty of Dentistry, Bingöl University, 12000 Bingöl, Türkiye; bktartuk@bingol.edu.tr; 2 Department of Endodontics, Faculty of Dentistry, Dicle University, 21280 Diyarbakır, Türkiye

**Keywords:** zirconia-reinforced lithium silicate, PEEK, endocrown, additive manufacturing, thermocycling, marginal adaptation, fracture resistance

## Abstract

The restoration of endodontically treated teeth remains a clinical challenge, particularly when substantial coronal tissue loss is present. Endocrowns fabricated using CAD/CAM technologies offer a conservative and esthetic alternative to conventional post-core systems; however, their long-term performance may be influenced by age-related mechanical and thermal stresses. This study evaluated the effect of thermomechanical aging on the marginal adaptation and fracture resistance of endocrowns fabricated from three CAD/CAM materials: zirconia-reinforced lithium silicate (ZLS), polyetherether ketone (PEEK), and 3D-printed resin. Sixty extracted human molars were endodontically treated and restored with endocrowns produced from these materials (*n* = 20 per group) and then subdivided into aged (*n* = 10) and control (*n* = 10) subgroups. Thermomechanical aging involved 5000 thermal cycles between 5 °C and 55 °C, and 75,000 mechanical loading cycles at 50 N. Marginal gaps were examined using scanning electron microscopy, and fracture resistance was tested under axial load at a crosshead speed of 0.5 mm/min. Data were analyzed using two-way ANOVA followed by Tukey’s post hoc test (α = 0.05). Thermomechanical aging significantly increased the marginal gaps in all materials (*p* < 0.05). The smallest marginal discrepancies were observed in the 3D-printed resin group, while the largest occurred in the ZLS after aging, likely due to dimensional changes during crystallization. Fracture resistance decreased in ZLS (−21.2%) and 3D resin (−20.9%) after aging (*p* < 0.05) but was not significantly affected in PEEK (−5.4%, *p* = 0.092). Thermomechanical aging adversely affects marginal adaptation across all materials, whereas its impact on strength is material-dependent. PEEK demonstrated the most stable mechanical performance and may represent a promising alternative for long-term endocrown restorations.

## 1. Introduction

The restoration of endodontically treated teeth remains a persistent clinical challenge in contemporary dentistry [1]. The substantial loss of hard tissue commonly observed in such teeth renders them more susceptible to functional stresses and structural compromise [2]. Therefore, re-establishing the structural integrity of these teeth is critical for ensuring a favorable long-term prognosis [3].

Traditionally, intraradicular post systems, particularly fiber posts, have been employed to enhance retention and improve stress distribution in teeth with extensive coronal loss [4,5]. However, current evidence indicates that the placement of a post alone does not significantly increase fracture resistance. Instead, treatment success largely depends on the amount of remaining coronal tooth structure and the effectiveness of adhesive bonding [6,7].

Advances in adhesive dentistry have shifted attention toward endocrown restorations, which provide a more conservative and esthetic alternative for the rehabilitation of endodontically treated teeth [8,9]. Endocrowns offer several advantages over conventional post-core systems, including improved fracture resistance, reduced chair time, ease of application, superior esthetics, and cost-effectiveness [2,10].

Progress in CAD/CAM technology has facilitated rapid and precise fabrication of fixed restorations with fewer manual steps [11,12]. This digital workflow not only shortens clinical procedures but also enhances marginal fit, internal adaptation, and overall esthetic quality [13,14]. The broader adoption of CAD/CAM systems has also expanded the range of compatible restorative materials [15].

Among these materials, zirconia-reinforced lithium silicate (ZLS) glass-ceramics have emerged as a prominent option for the fabrication of endocrowns. Although ZLS provides an advantageous balance between esthetics and mechanical properties, glass ceramics inherently exhibit brittleness and may contribute to excessive antagonist occlusion [16,17].

To overcome these limitations, polyetherether ketone (PEEK), a high-performance polymer with a favorable elastic modulus and reduced brittleness, has been proposed. Owing to its biomechanical compatibility, PEEK is considered a promising material for posterior restorations [18].

While subtractive CAD/CAM milling offers high precision, reduced technician-dependent variability, and reliable quality control, it also presents drawbacks, such as microcrack formation, surface irregularities, and material waste [19,20]. To mitigate these disadvantages, additive manufacturing technologies, particularly 3D printing, have gained traction in restorative dentistry, enabling efficient fabrication and improved material utilization [19,21].

The long-term clinical success of indirect restorations largely depends on esthetic quality and marginal adaptation [22]. Inadequate marginal fit can lead to microleakage, secondary caries, plaque accumulation, and ultimately restoration failure [23]. Although a marginal gap threshold of 120 μm is commonly cited as clinically acceptable, a universally defined standard is still lacking [24].

Another determinant of clinical performance is fracture resistance, which reflects the restorative material’s ability to withstand masticatory forces and oral stresses [25]. While catastrophic failures may be uncommon in the short term, their incidence increases following aging-related degradation [26]. Thermomechanical aging is widely used as a reliable in vitro method to simulate long-term clinical service conditions and evaluate restorative performance [27,28,29].

Despite the growing interest in endocrown restorations, data regarding the combined effects of manufacturing technique, material type, and thermomechanical aging on restorative performance remain limited. In particular, direct comparisons between milled CAD/CAM materials and additively manufactured (3D-printed) resins under standardized preparation and aging conditions are scarce. Moreover, existing studies often focus on either marginal adaptation or fracture resistance alone, resulting in inconsistent or insufficient evidence regarding the long-term behavior of endocrown restorations.

Therefore, the novelty of the present study lies in the simultaneous evaluation of marginal fit and fracture resistance of endocrown restorations fabricated from different materials using both subtractive CAD/CAM milling and additive manufacturing (3D printing) techniques, following thermomechanical aging under strictly standardized experimental conditions. This comprehensive approach provides clinically relevant insight into the long-term performance of contemporary endocrown materials.

The aim of this in vitro study was to evaluate the effect of thermomechanical aging on the marginal fit and fracture resistance of endocrown restorations fabricated using different CAD/CAM materials and manufacturing techniques. The null hypothesis (H_0_) stated that thermomechanical aging would have no significant effect on the marginal adaptation or fracture resistance of endocrowns fabricated from different CAD/CAM materials.

## 2. Materials and Methods

### 2.1. Study Design

The materials used in this study are listed in Table 1. This in vitro experiment was conducted at the Faculty of Dentistry, Dicle University, with approval from the Institutional Ethics Committee (Approval No: 2025-82).

**Figure 1:**
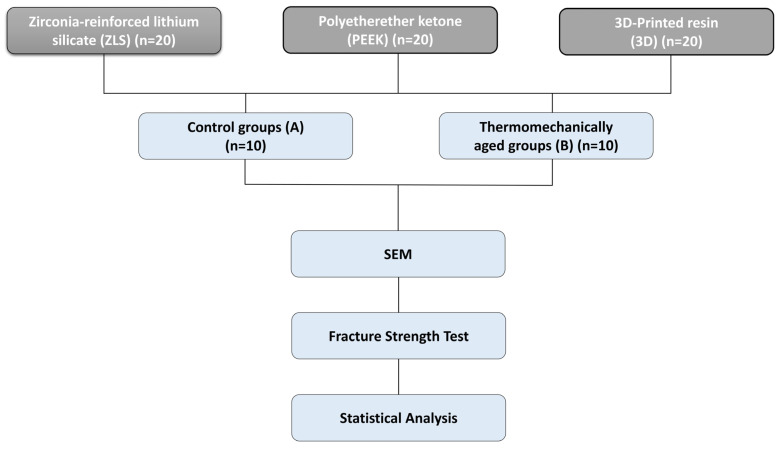
Study workflow illustrating the experimental design for the evaluation of CAD/CAM endocrown materials.

Sample size estimation was performed using G*Power v3.1.9.6. The primary outcome variable was fracture resistance (N) and the secondary outcome variable was marginal gap (µm). The calculation was based on a two-way ANOVA design (three materials × two aging conditions; α = 0.05) with a power of 1 − β = 0.95. An effect size of f = 0.40 (medium–large) was selected based on preliminary data obtained from our laboratory. Under these assumptions, the required total sample size was determined to be *n* = 60 (*n* = 10 per subgroup), which were included in the experiment (Figure 1).

### 2.2. Specimen Selection and Standardization

After obtaining informed consent, 60 sound, extracted human mandibular first molars with fully developed roots and no caries or cracks were selected. The teeth were extracted for periodontal reasons and stored for no longer than 30 days in a 0.1% thymol–0.9% NaCl solution at room temperature. The inclusion criteria were teeth with similar buccolingual and mesiodistal dimensions, a minimum pulp chamber depth of 2 mm, and no calcifications or structural defects.

Dimensional standardization was verified by measuring the mesiodistal and buccolingual widths at the cementoenamel junction using a digital caliper (150 mm Digital Caliper; Mitutoyo Corp., Kawasaki, Japan). Specimens deviating by more than 10% were excluded. Residual periodontal ligament and calculus were manually removed using hand scalers (Hu-Friedy; Mfg. Co., LLC, Chicago, IL, USA).

### 2.3. Endodontic Treatment

All the endodontic procedures were performed by a single experienced endodontist. Conventional root canal treatment was performed. Access cavities were prepared using water-cooled diamond burs (Medium-grit, round diamond burs; Komet Dental GmbH, Lemgo, Germany). Root canal shaping was completed using ProTaper Next rotary files (X1–X2; Dentsply Sirona, Charlotte, NC, USA), according to the manufacturer’s instructions. The working length was determined radiographically and set 1 mm short of the apical foramen. Each canal was irrigated with 3 mL 5.25% sodium hypochlorite (NaOCl) and 2 mL 17% EDTA during instrumentation.

After drying with sterile paper points, the canals were obturated using the single-cone technique with F3 gutta-percha (Dentsply Sirona; Konstanz, Germany) and ADSEAL sealer (Meta Biomed Co., Ltd., Cheongju, Republic of Korea). Excess gutta-percha was removed and vertically compacted using a heated plugger (Woodpecker Fi-P; Guilin, China). The access cavities were sealed with a 2 mm layer of flowable composite resin (Nexcomp A2; Meta Biomed Co., Ltd., Cheongju, Republic of Korea) following the application of a thin coat of All-Bond Universal adhesive to enhance bonding. All polymerization procedures were performed using an LED light-curing unit (DTE O-Light; Woodpecker, Guilin, China) with an output of 650 mW/cm^2^.

### 2.4. Endocrown Fabrication

**Figure 2:**
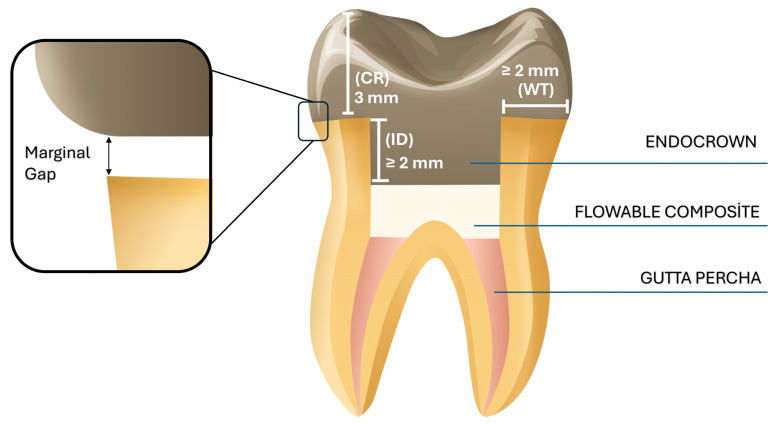
Illustrative view of the prepared tooth for an endocrown restoration. WT: dentine wall thickness; CR: cervical reduction; ID: intracoronal depth of the pulp chamber extension.

Standardized endocrown preparations were performed with a diamond bur (Medium-grit, rounded-end tapered diamond bur; Komet Dental GmbH, Lemgo, Germany), maintaining a 1 mm supragingival margin above the cementoenamel junction. Preparations were standardized using a 5-axis CNC milling system (Premium4820 imes-icore; GmbH, Eiterfeld, Germany) under continuous water cooling, achieving an internal divergence of 8–10°. The mean cavity depth was standardized at 3 mm and verified using a digital caliper (Figure 2). The internal surfaces were refined with a cylindrical diamond bur (Fine-grit cylindrical diamond bur; Komet Dental, GmbH, Lemgo, Germany) and polished using flexible aluminum oxide abrasive disks (Sof-Lex™ Pop-On; 3M ESPE, St. Paul, MN, USA) to ensure uniform cement thickness.

The specimens were randomly assigned to three experimental groups (*n* = 20 each):

Group ZLS–Zirconia-reinforced lithium silicate glass-ceramic endocrowns.

Group PEEK–Polyetherether ketone (PEEK) endocrowns.

Group 3D–3D-printed resin endocrowns.

**Figure 3:**
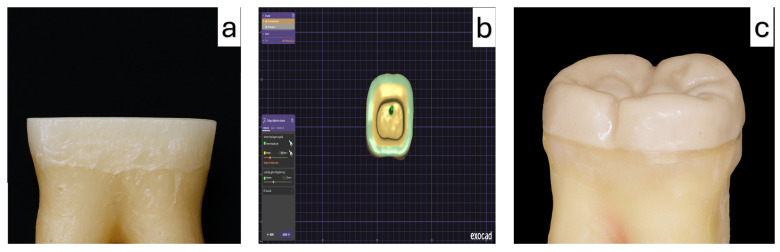
Endocrown preparation (**a**), computer-aided design of the restoration using CAD software (**b**), and the final PEEK endocrown restoration after fabrication (**c**).

Each specimen was scanned using an intraoral scanner (Trios 4; 3Shape, Copenhagen, Denmark), and digital data were exported in standard tessellation language (STL) format (*n* = 60 models). Endocrowns were designed using Exocad software (v3.0; Exocad GmbH, Darmstadt, Germany) with a uniform cement space of 50 µm and standardized occlusal height of 5 mm (Figure 3).

ZLS and PEEK restorations were fabricated via 5-axis milling, inspected for defects, and polished according to the manufacturer’s protocols. Three-dimensional resin endocrowns were printed using a Stereolithography Apparatus (SLA-based) Formlabs 3D printer (Formlabs Inc., Somerville, MA, USA) and post-cured under 405 nm UV light for 30 min using a Form Cure unit. A permanent crown resin (Crowntec^®^; Saremco Dental AG, Rebstein, Switzerland), composed primarily of bisphenol A polyethylene glycol diether dimethacrylate (BisEMA), with methyl benzoylformate and a TPO-type photoinitiator, was used for fabrication.

### 2.5. Cementation Procedure

All endocrown restorations were ultrasonically cleaned in 99% isopropanol for 3 min using an ultrasonic cleaner (Ultrasonic Cleaner UC-20; Biobase, Jinan, China) and rinsed with distilled water. For ZLS restorations, the internal surfaces were etched with 5% hydrofluoric acid gel (IPS Ceramic Etching Gel, Ivoclar Vivadent AG, Schaan, Liechtenstein) for 20 s, rinsed thoroughly, and air-dried. A silane coupling agent (Monobond S; Ivoclar Vivadent AG, Schaan, Liechtenstein) was applied for 60 s and gently air-dried.

The intaglio surfaces of PEEK restorations were airborne-particle abraded with 50 µm Al_2_O_3_ particles (Bego Korrocoat, Bremen, Germany) at 2 bar pressure for 10 s and cleaned with oil-free air. A universal bonding primer (Visio.link; Bredent GmbH, Senden, Germany) was used. The internal surfaces of the 3D-printed resin restorations were conditioned using the same airborne-particle abrasion protocol, followed by ultrasonic cleaning in ethanol for 3 min (Ultrasonic Cleaner UC-20, Biobase) and air drying.

The tooth surfaces were etched with 37% phosphoric acid (Scotchbond Universal Etchant; 3M ESPE, St. Paul, MN, USA) for 15 s, rinsed, and then an adhesive layer (All-Bond Universal, Bisco Inc., Schaumburg, IL, USA) was applied. All restorations were cemented using a dual-cure resin cement (Variolink Esthetic DC; Ivoclar Vivadent AG, Schaan, Liechtenstein). The excess cement was removed before polymerization, and the marginal areas were subsequently finished and polished using flexible abrasive disks (Sof-Lex™; 3M ESPE, St. Paul, MN, USA).

Following cementation, all the specimens were stored in distilled water at room temperature. Ten randomly selected specimens from each group (*n* = 10) were assigned to the aging subgroup, whereas the remaining specimens served as the non-aged control group.

### 2.6. Thermomechanical Aging

For thermal aging, the restorations were subjected to 5000 thermal cycles between 5 and 55 °C, with a dwell time of 25 s in each bath and a transfer time of 10 s. Mechanical aging was performed following thermal cycling using a chewing simulator (Robota Chewing Simulator, Model ACH-09075DCT; AD-Tech Technology Co., Ltd., Lübeck, Germany). During fatigue cycling, the samples were maintained under temperature-controlled conditions at 37 °C to simulate the intraoral environment. Each specimen was loaded with an occlusal force of 50 N, representing the average physiological load in patients without bruxism, at a frequency of 1.6 Hz for 75,000 cycles. These parameters approximate the clinical equivalent of approximately six months to one year of masticatory activity.

After completion of the aging procedures, the specimens were stored in isotonic saline at room temperature 24 h prior to marginal gap evaluation. To simulate periodontal ligament (PDL) behavior, each root surface was coated with a 0.2–0.3 mm layer of vinyl polysiloxane-based elastomer (Elite HD+; Zhermack, Badia Polesine, Rovigo, Italy). Following the PDL simulation, the teeth were vertically embedded in self-curing polyvinyl chloride (PVC) cylinders positioned 2 mm below the cementoenamel junction to provide a more clinically realistic stress distribution during testing.

### 2.7. Marginal Gap Measurement

Marginal adaptation was evaluated based on the vertical marginal discrepancy between the restoration and tooth preparation margin. Measurements were performed using a scanning electron microscope (SEM). Prior to imaging, all specimens were sputter-coated with a ~10 nm layer of gold–palladium using a sputter coater (Emitech K550 Sputter Coater; Quorum Technologies Ltd., Lewes, UK) to ensure adequate surface conductivity and prevent charging artifacts under high vacuum conditions.

**Figure 4:**
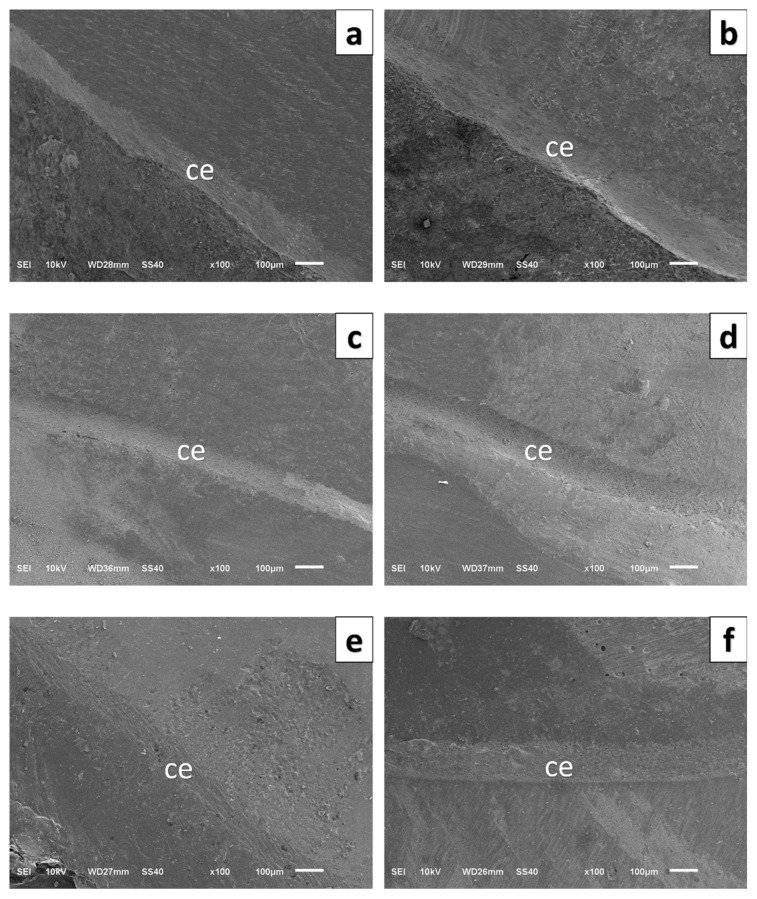
High-magnification scanning electron microscopy images of the border between crown, cement, and tooth structure. (**a**) PEEK-CG, (**b**) PEEK–TG, (**c**) 3D-printed resin–CG, (**d**) 3D-printed resin–TG, (**e**) ZLS–CG, (**f**) ZLS–TG.CG: Control Group, TG: Thermomechanically aged group, ce: Cement.

For each specimen, a total of 20 marginal gap measurements were recorded, with five points taken from each of the four axial surfaces (buccal, lingual, mesial, and distal), following a method widely accepted for its accuracy. SEM imaging was performed using a JEOL JSM-6510 SEM (JEOL Ltd., Tokyo, Japan) at an accelerating voltage of 10 kV. Before measurement, the device was calibrated using a certified 10 µm reference standard. All measurements were conducted at 100× magnification by a single examiner experienced in SEM analysis to minimize examiner-related variability. The mean value of the 20 measurements obtained for each specimen was used as the marginal gap value (Figure 4).

To assess measurement repeatability, ten randomly selected specimens were re-measured, and intra-observer reliability was calculated using the intraclass correlation coefficient (ICC), which was found to be ≥0.90. Results are expressed as the mean ± standard deviation (µm).

### 2.8. Fracture Resistance Test

All specimens were subjected to axial compressive loading using a universal testing machine equipped with a 10 kN load cell Shimadzu Model AGS-X (Shimadzu Corporation, Kyoto, Japan). All tests were conducted at room temperature (approximately 23 ± 2 °C). The load was applied axially and centrally to the deepest point of the occlusal groove using a 5 mm diameter spherical stainless-steel indenter at a crosshead speed of 0.5 mm/min. To reduce stress concentration, a 0.5 mm thick elastomeric interface (polyurethane sheet) was placed between the indenter and occlusal contact point, ensuring a more physiologic distribution of load across the tooth–restoration complex. The maximum load at the moment of fracture was recorded in Newtons (N) and reported for each group as mean ± standard deviation (SD).

### 2.9. Statistical Analysis

All statistical analyses were performed using IBM SPSS Statistics version 20.0 (IBM Corp., Armonk, NY, USA). The normality of the data was assessed using the Shapiro–Wilk test, and homogeneity of variances was evaluated using Levene’s test. Both assumptions were satisfied for all the groups. The Shapiro–Wilk test indicated that the marginal gap and fracture resistance data were normally distributed in all groups (*p* > 0.05); therefore, parametric statistical analyses were applied.

The marginal gap and fracture resistance values were analyzed based on two independent factors (restorative material and thermomechanical aging). Accordingly, separate two-way analyses of variance (Two Way ANOVA) were conducted for each dependent variable. When significant main effects or interactions were detected, pairwise group comparisons were performed using Tukey’s honest significant difference post hoc test. A significance level of *p* < 0.05 was adopted for all analyses.

## 3. Results

No premature restoration fractures were observed in any specimen during the thermomechanical aging process. Therefore, all endocrowns were subjected to static loading tests until failure occurred. Normality assumptions were met (Shapiro–Wilk, *p* > 0.05); therefore, results are presented as mean ± SD and analyzed using two-way ANOVA.

### 3.1. Marginal Gap Analysis

The mean marginal gap values of the three groups were compared to control groups and thermomechanically aged groups. The lowest mean marginal gap was observed in the 3D-printed resin control group (67.08 ± 11.22 µm), while the highest mean value after aging was recorded in the ZLS-aged group (102.02 ± 45.60 µm) (Table 2).

**Figure 5:**
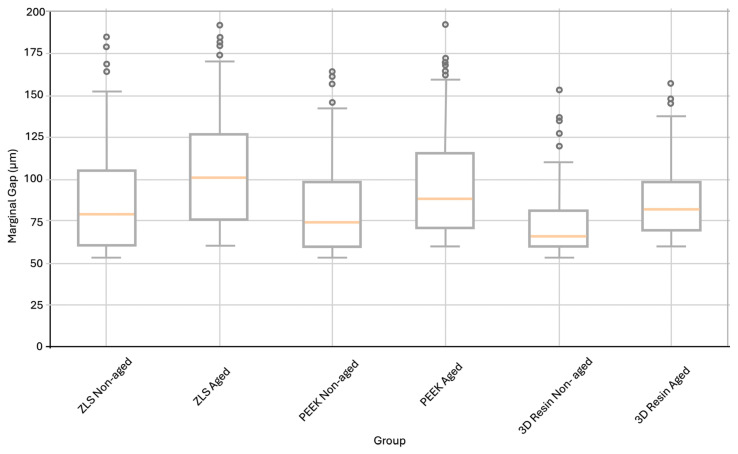
Distribution of marginal gap values (µm) for ZLS, PEEK, and 3D-printed resin endocrowns in non-aged and thermomechanical aged conditions, presented as box plots to illustrate data dispersion and central tendency, although normality assumptions were met. The box represents the interquartile range, the horizontal line inside the box indicates the median, the whiskers denote the minimum and maximum values within 1.5× the interquartile range, and the circles represent outliers.

Statistical analysis revealed that thermomechanical aging significantly increased the marginal gap values for all the materials (*p* < 0.05). The marginal gap increased by 25.8, 19.5, and 23.9% in the ZLS, PEEK, and 3D-printed resin groups, respectively. This increase was statistically significant for all three materials (ZLS, *p* = 0.012; PEEK, *p* = 0.021; 3D resin, *p* = 0.018). As marginal gap evaluation involved a large number of individual measurements per group, box plot analysis was used to visually present data dispersion, median values, interquartile ranges, and potential outliers (Figure 5). Although the data met the assumptions of normality (Shapiro–Wilk test, *p* > 0.05) and were analyzed using parametric methods, box plots were preferred as a complementary graphical tool to facilitate a clear comparison of marginal adaptation among different materials before and after thermomechanical aging.

### 3.2. Fracture Resistance

The main effect of the material type on the fracture resistance was statistically significant (*p* < 0.05). The main effect of aging was also significant, but its magnitude varied among materials. A significant material × aging interaction was detected, indicating that the influence of aging on the fracture strength was material-dependent.

**Figure 6:**
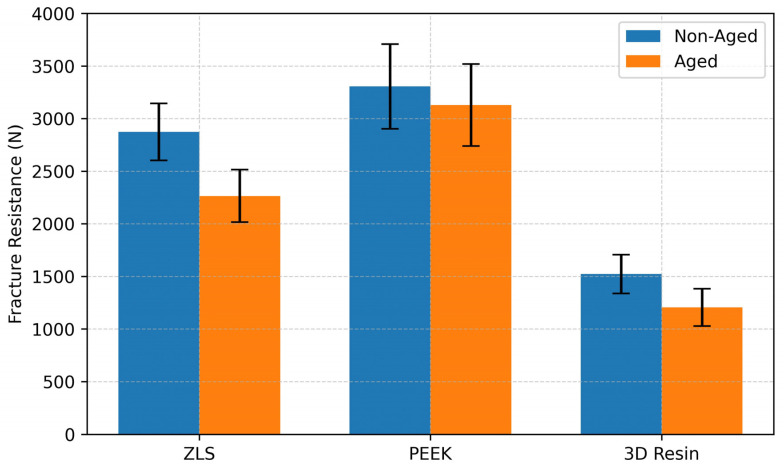
Comparison of mean fracture resistance values (N) for ZLS, PEEK, and 3D resin endocrowns in non-aged and thermomechanically aged conditions. Error bars indicate standard deviation.

For the non-aged specimens, mean fracture resistance values were as follows: ZLS: 2874.21 ± 211.57 N; PEEK: 3306.78 ± 401.88 N; 3D resin: 1525.62 ± 184.09 N. After thermomechanical aging, the values decreased to 2265.32 ± 349.74 N, 3129.53 ± 389.14 N, and 1205.48 ± 176.42 N, respectively (Table 3, Figure 6). The percentage reduction in fracture resistance following aging was 21.2% for ZLS, 5.4% for PEEK, and 20.9% for the 3D resin.

**Figure 7:**
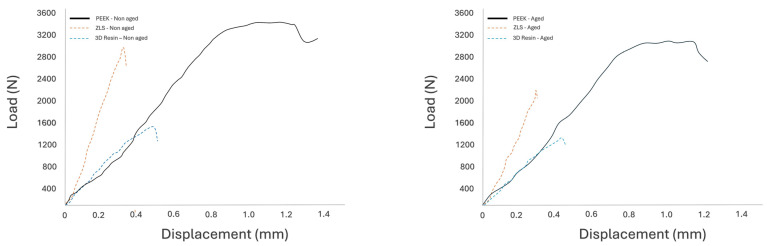
Representative load–displacement curves for the three tested endocrown materials.

Representative force–displacement curves (Figure 7) further illustrated differences in mechanical behavior among the tested materials. PEEK exhibited a more gradual load increase with greater displacement prior to fracture, indicating higher deformation capacity, whereas ZLS and 3D resin showed steeper load–displacement slopes and earlier fracture, reflecting a more brittle response.

The reduction was statistically significant for ZLS (*p* = 0.004) and 3D resin (*p* = 0.003) but not for PEEK (*p* = 0.092). Post hoc multiple comparison tests revealed significant differences among the aged groups: PEEK–ZLS, PEEK–3D resin, and ZLS–3D resin (*p* < 0.001) (Table 4).

## 4. Discussion

In this in vitro study, the fracture resistance and marginal adaptation of molar endocrown restorations fabricated using different CAD/CAM materials and manufacturing techniques were evaluated before and after the chewing simulation. Following thermomechanical aging, statistically significant changes were observed in both the marginal fit and fracture resistance; therefore, the null hypothesis was rejected. These findings are consistent with previous studies reporting that thermomechanical aging has a direct impact on the structural durability of restorative materials [26,30].

The use of natural human molar teeth enables a more clinically realistic simulation of functional behavior due to inherent variations in hard tissue properties and anatomical structures. Although all specimens were standardized using a CNC-controlled milling system, complete uniformity could not be achieved because of the biological variability in dentin density, tubule configuration, and canal morphology, which may have contributed to the observed data variations [31,32].

CAD/CAM technology has been utilized to achieve greater control over the restoration thickness, internal geometry, and marginal integrity [33]. The 5-axis milling system used in this study facilitated enhanced internal adaptation, particularly in restorations with complex morphology [31]. Because the burr diameter and geometry can directly influence the marginal fit, the manufacturing procedures were strictly standardized [34].

There is no universally accepted method for evaluating the marginal adaptation. Among the commonly used techniques is the measurement of the marginal gap, defined as the space between the restoration margin and the tooth surface. Various in vitro approaches have been described in the literature, including direct microscopic evaluation, sectioning techniques, and silicone replica methods [35,36]. In this study, SEM was preferred because it is non-destructive, allows repeated measurements before and after aging, and provides multiple evaluation points around each restoration, thereby minimizing errors from regional variability.

During the cementation process, finger pressure was applied to simulate the clinical conditions. This method has also been adopted in previous in vitro studies, such as that by Att et al. Although the inability to standardize finger pressure remains a potential limitation owing to operator-dependent variability [28].

To simulate the supporting bone, the specimens were embedded in a self-curing epoxy resin with an elastic modulus (approximately 18 GPa) similar to that of the human cortical bone. Additionally, during fracture testing, a load was applied along the long axis of each restoration to standardize the stress distribution [37].

In the non-aged groups, the lowest marginal gap values were observed in the 3D-printed resin group, and the highest values were observed in the ZLS group. This may be attributed to the dimensional changes occurring during the crystallization firing of ZLS ceramics, which may negatively influence marginal adaptation [38]. The superior marginal fit of the 3D-printed group can be explained by the selected printing parameters, particularly the 50 µm layer thickness, allowing high-resolution reproduction of fine details. In contrast, the milled groups may have exhibited reduced adaptation due to the limited detailed reproduction capability of the burs used (1.8 mm tip diameter). The incremental layer-by-layer fabrication inherent in 3D printing also supports accurate geometry reproduction and may help compensate for polymerization shrinkage effects [39].

The clinically acceptable threshold for marginal gaps is commonly reported to be 120 µm [40]. All marginal gap values obtained in this study remained well below this limit in both the non-aged and thermomechanically aged groups, indicating clinically acceptable marginal integrity across all materials.

No statistically significant differences were found between the three material groups when the effects of thermomechanical aging on marginal adaptation were compared. This finding aligns with studies demonstrating no significant difference in marginal adaptation between 3D-printed resin crowns and milled ceramic crowns [41]. Although the PEEK endocrowns did not differ significantly from the ZLS group in marginal and internal adaptation, they demonstrated superior mean values, consistent with previous reports [42,43].

A significant increase in the marginal gap values was observed in all the material groups following thermomechanical aging. This may be associated with the micromechanical degradation induced by thermal stresses. Previous studies have attributed such changes to accelerated hydrolysis of exposed collagen fibers, deterioration of inadequately polymerized resin components after water exposure, and differences in thermal expansion coefficients between the tooth structure and restorative materials, leading to stress accumulation at the interface [44]. Thermal cycling has also been reported to induce dimensional changes that initiate microcrack formation within restorative materials [45,46], whereas similar studies have reported increased marginal gaps after aging across all restoration types [47]. In contrast, Eldin et al. reported no significant influence of thermomechanical aging on marginal adaptation, although they observed reduced fracture resistance, irrespective of the material type [37].

Variability among studies often arises from methodological differences, including restoration type (crown, inlay, onlay, endocrown), material properties, manufacturing method (milling vs. 3D printing), scanning and milling accuracy, cement space settings, burr diameter, and measurement technique. These factors limit the direct comparability of results across studies.

Fracture and crack formation are among the most common complications associated with the long-term clinical use of dental ceramics. Therefore, this study also evaluated the fracture resistance of CAD/CAM endocrowns in both the non-aged and thermomechanically aged groups under simulated clinical conditions. A previous investigation reported that fractures in long-term ceramic restorations often originate from adjacent regions affected by compressive stresses and not from the occlusal contact zone itself [48]. Accordingly, vertical loading was applied to the occlusal surface to more closely simulate the clinical stress distribution.

All material groups withstood forces exceeding the physiological occlusal load range and met the basic clinical durability criteria. However, thermomechanical aging resulted in a statistically significant reduction in fracture resistance, particularly in the ZLS and 3D-printed resin groups (*p* < 0.001). This reduction may be attributed to microstructural characteristics and varying sensitivities to heat and moisture exposure.

PEEK restorations exhibited the highest fracture resistance values, followed by ZLS and 3D-printed resins. The enhanced performance of PEEK may be related to its precise fabrication process and ability to provide superior internal adaptation. Villefort et al. reported that PEEK materials demonstrated high compressive strength and impact absorption capacity, resulting in lower stress concentrations within restorations [49]. The biomechanical properties of PEEK (compressive strength 246 MPa, elastic modulus 5.1 GPa) are similar to those of dentin (297 MPa, 18.6 GPa), supporting the stress compatibility between the restoration and tooth structure [50].

The ZLS exhibited a significantly higher fracture resistance than the 3D-printed resin. This supports the superior mechanical performance of ZLS ceramics, attributed to their polydispersed crystal structure with nano-zirconia reinforcement, which promotes crack deflection and inhibits propagation [51,52]. The uniform fine-grained microstructure facilitates a balanced stress distribution and contributes to a higher fracture resistance [37].

Although all groups exhibited reduced fracture resistance after aging, only the ZLS and 3D resin groups showed a statistically significant decrease. Thermal mismatch between restorative materials and tooth structures results in stress accumulation at the interface, promoting microcrack initiation and propagation, particularly in brittle materials [53,54]. The viscoelastic nature of PEEK may explain why its reduction in fracture resistance was not statistically significant; its flexible matrix allows more elastic deformation under thermomechanical loads, thereby reducing crack initiation [55].

In contrast, the 3D-printed resin group exhibited pronounced mechanical degradation after aging. Resin-based materials are susceptible to water absorption, leading to hydrolytic weakening at the matrix–filler interface. SLA-printed resins, with their layered microstructure and limited cross-link density, are particularly prone to structural deterioration during thermal cycling [56].

Overall, the findings indicate that the effects of thermomechanical aging on fracture resistance are material dependent. As long-term clinical success relies on restorations maintaining their mechanical performance under continuous intraoral stress, evaluating their post-aging behavior is critical for predicting clinical longevity [57]. Few studies have simultaneously compared milled and 3D-printed materials in terms of fracture resistance after thermomechanical aging [58]. Therefore, the present study contributes valuable comparative data regarding the durability of endocrowns fabricated from PEEK, ZLS, and 3D-printed resins.

Certain limitations of this study must be considered when interpreting these findings. Fracture resistance was assessed only under vertical loading, whereas intraoral forces are multidirectional; lateral and shear stresses may influence the mechanical behavior of restorations differently. In addition, in vitro conditions can simulate the oral environment only to a limited extent.

Another limitation is related to the aging protocol, as thermal and mechanical aging were applied successively rather than simultaneously. This sequential approach was intentionally selected because it is widely used in in vitro studies and allows greater experimental control by enabling the isolated assessment of thermal and mechanical degradation mechanisms. However, under clinical conditions, endocrown restorations are exposed to coupled thermomechanical aging, in which temperature fluctuations and occlusal loading occur concurrently. Since temperature can significantly influence mechanical fatigue behavior, particularly in resin-based materials, this methodological choice may not fully replicate intraoral aging conditions and should therefore be considered a limitation when interpreting the results.

Furthermore, the marginal gap evaluation was performed using scanning electron microscopy, and sample preparation procedures such as dehydration and exposure to vacuum conditions may potentially contribute to artificial gap formation or interfacial debonding, particularly in resin-based materials. Although all specimens were subjected to the same standardized SEM preparation protocol, calibration procedure, and imaging conditions, SEM-induced artifacts cannot be completely excluded. Therefore, this factor should be regarded as an additional limitation when interpreting marginal gap measurements.

Consequently, long-term in vivo studies employing multidirectional loading conditions and clinically relevant evaluation methods are required to validate the clinical applicability of the present findings.

## 5. Conclusions

Within the limitations of this in vitro study, all groups demonstrated fracture resistance and marginal adaptation values within the ranges reported in laboratory-based studies. Thermomechanical aging caused a significant increase in marginal gap values across all groups and negatively affected the performance of glass-ceramic and resin-based materials, in particular.

These findings suggest that PEEK may be a durable and biomechanically compatible endocrown option for endodontically treated posterior teeth. Although ZLS exhibits high initial strength, its performance decreases with aging. Despite demonstrating good initial adaptation and low production costs, 3D-printed resins should be approached with caution because of their susceptibility to mechanical degradation following thermomechanical stress.

## Figures and Tables

**Table 1:** Materials used in this study.

Material	Product Name	Composition	Manufacturer
Zirconia-reinforced lithium silicate glass-ceramic (ZLS)	Vita Suprinity^®^ PC	SiO_2_: 56–64% Li_2_O: 15–21% ZrO_2_: 8–12% TiO_2_: ~10% Coloring pigments: <10%	VITA Zahnfabrik H. Rauter GmbH, Bad Säckingen, Germany
3D-printed Resin	Crowntec^®^	BisEMA (Bisphenol A polyethylene glycol diether dimethacrylate): 50–<75% Methyl benzoylformate: 1–<5% TPO-type photoinitiator: 1–<5%	Saremco Dental AG, Rebstein, Switzerland
Polyetherether ketone (PEEK)	Ceramill^®^ PEEK	Polyetheretherketone ~100% high-purity, unfilled polymer	Amann Girrbach, Mäder, Austria

**Table 2:** Mean, standard deviation SD (±) values (µm), and percentage change in vertical marginal gap among different control groups and thermomechanically aged groups.

Material	Control Groups (A) (*n* = 10)	Thermomechanically Aged Groups (B) (*n* = 10)	% Change (B–A)	F Test	*p* Value
ZLS	81.21 ± 38.78	102.02 ± 45.60	+25.8	6.784	0.012 *
PEEK	74.19 ± 24.56	88.67 ± 39.74	+19.5	5.319	0.021 *
3D Resin	67.08 ± 11.22	83.38 ± 33.96	+23.9	5.911	0.018 *

* Significant differences are indicated.

**Table 3:** Mean, standard deviation SD (±) values (Newton), and percentage change in the fracture resistant different control group and thermomechanically aged group.

Material	Control Groups (A) (*n* = 10)	Thermomechanically Aged Groups (B) (*n* = 10)	% Change (B–A)	F Test	*p* Value
ZLS	2874.21 ± 271.57	2265.32 ± 249.74	−21.2	8.752	0.004 *
PEEK	3306.78 ± 401.88	3129.53 ± 389.14	−5.4	2.187	0.092
3D Resin	1523.62 ± 184.09	1205.48 ± 176.42	−20.9	9.416	0.003 *

* Significant differences are indicated.

**Table 4:** After, thermomechanical aging were statistically compared using multiple comparison analysis of fracture resistant.

Material	*p* Value
ZLS-PEEK	0.0000049 *
PEEK–3D Resin	0 *
ZLS-3D Resin	0 *

* Significant differences are indicated.

## Data Availability

The original contributions presented in this study are included in the article. Further inquiries can be directed to the corresponding author.

## Abbreviations

The following abbreviations are used in this manuscript:

3D	Three-Dimensional
°C	Degree Celsius
ANOVA	Analysis of Variance
BisEMA	Bisphenol A ethoxylate dimethacrylate
CAD/CAM	Computer-Aided Design / Computer-Aided Manufacturing
EDTA	Ethylenediaminetetraacetic Acid
ICC	Intraclass Correlation Coefficient
NaOCl	Sodium Hypochlorite
N	Newton
PDL	Periodontal Ligament
PEEK	Polyetherether Ketone
PVC	Polyvinyl Chloride
SD	Standard Deviation
SEM	Scanning Electron Microscopy
SLA	Stereolithography Apparatus
ZLS	Zirconia-Reinforced Lithium Silicate
µm	Micrometer
